# Deep Gene Sequence Cluster Analyses of Multi-Virus-Infected Mucosal Tissue Reveal Enhanced Transmission of Acute HIV-1

**DOI:** 10.1128/JVI.01737-20

**Published:** 2021-01-13

**Authors:** Katja Klein, Immaculate Nankya, Gabrielle Nickel, Annette N. Ratcliff, Adam A. J. Meadows, Nicholas Hathaway, Jeffrey A. Bailey, Daniel J. Stieh, Hannah M. Cheeseman, Ann M. Carias, Michael A. Lobritz, Jamie F. S. Mann, Yong Gao, Thomas J. Hope, Robin J. Shattock, Eric J. Arts

**Affiliations:** a Department of Microbiology and Immunology, University of Western Ontario, London, Ontario, Canada; b Joint Clinical Research Centre, Kampala, Uganda; c Department of Medicine, Case Western Reserve University, Cleveland, Ohio, USA; d Department of Medicine, University of Massachusetts Medical School, Worcester, Massachusetts, USA; e Warren Alpert Medical School, Brown University, Providence, Rhode Island, USA; f Department of Cellular and Molecular Medicine, St. George’s, University of London, London, United Kingdom; g Department of Medicine, Imperial College London, London, United Kingdom; h Department of Cellular and Molecular Biology, Feinberg School of Medicine, Northwestern University, Chicago, Illinois, USA; Icahn School of Medicine at Mount Sinai

**Keywords:** HIV, fitness, transmission

## Abstract

Exposure of the genital mucosa to a genetically diverse viral swarm from the donor HIV-1 can result in breakthrough and systemic infection by a single transmitted/founder (TF) virus in the recipient. The highly diverse HIV-1 envelope (Env) in this inoculating viral swarm may have a critical role in transmission and subsequent immune response. Thus, chronic (Env_chronic_) and acute (Env_acute_) Env chimeric HIV-1 were tested using multivirus competition assays in human mucosal penile and cervical tissues. Viral competition analysis revealed that Env_chronic_ viruses resided and replicated mainly in the tissue, while Env_acute_ viruses penetrated the human tissue and established infection of CD4^+^ T cells more efficiently. Analysis of the replication fitness, as tested in peripheral blood mononuclear cells (PBMCs), showed similar replication fitness of Env_acute_ and Env_chronic_ viruses, which did not correlate with transmission fitness in penile tissue. Further, we observed that chimeric Env viruses with higher replication in genital mucosal tissue (chronic Env viruses) had higher binding affinity to C-type lectins. Data presented herein suggest that the inoculating HIV-1 may be sequestered in the genital mucosal tissue (represented by chronic Env HIV-1) but that a single HIV-1 clone (e.g., acute Env HIV-1) can escape this trapped replication for systemic infection.

**IMPORTANCE** During heterosexual HIV-1 transmission, a genetic bottleneck occurs in the newly infected individual as the virus passes from the mucosa, leading to systemic infection with a single transmitted HIV-1 clone in the recipient. This bottleneck in the recipient has just been described (K. Klein et al., PLoS Pathog 14:e1006754, https://doi.org/10.1371/journal.ppat.1006754), and the mechanisms involved in this selection process have not been elucidated. However, understanding mucosal restriction is of the utmost importance for understanding dynamics of infections and for designing focused vaccines. Using our human penile and cervical mucosal tissue models for mixed HIV infections, we provide evidence that HIV-1 from acute/early infection, compared to that from chronic infection, can more efficiently traverse the mucosal epithelium and be transmitted to T cells, suggesting higher transmission fitness. This study focused on the role of the HIV-1 envelope in transmission and provides strong evidence that HIV transmission may involve breaking the mucosal lectin trap.

## Abstract (precis)

During heterosexual HIV-1 transmission, a genetic bottleneck occurs in the newly infected individual as the virus passes from the mucosa, leading to systemic infection with a single transmitted HIV-1 clone in the recipient. This bottleneck in the recipient has just been described, and the mechanisms involved in this selection process have not been elucidated.

## INTRODUCTION

In the global spread of human immunodeficiency virus type 1 (HIV-1), the virus is most commonly transmitted during sexual intercourse. For this transmission event, the inoculating virus in vaginal fluids or semen must penetrate physical barriers, such as the mucus and epithelium of the genital mucosal tissue, to establish a propagating infection in susceptible target cells located in the underlying stroma. Regardless of the genital tissue, the risk of HIV-1 transmission per coital act is very low for receptive (0.08%) and insertive (0.04%) penile-vaginal intercourse (1), which is one factor associated with the higher and increasing HIV prevalence in women (2).

Over the years, multiple studies have indicated that a primary HIV-1 infection is initiated by one clone or a small number of transmitted/founder (TF) HIV-1 clones despite the presence of a diverse HIV-1 population in the donor (3, 4). Previous studies have examined the genetic bottlenecks from donor to recipient by describing the HIV in the blood of donors and recipients following transmission. However, there is considerable complexity in these transmission bottlenecks, in that the virus migrates and compartmentalizes in different tissues within the donor. For example, in a female-to-male HIV-1 transmission event, the virus within the female genital tract (FGT) could have been present since the time of acute infection. It is therefore possible that the virus could have evolved in the FGT or likely entered the FGT via the blood. Thus, the HIV-1 that is transmitted from the FGT to the penile tissue in female-to-male transmission may reflect what is transmitted from the vaginal mucosa rather than directly from the blood, in the absence of injury. The virus that infects the penile tissue may have originated from infection of the foreskin, urethra, or the glans tissue and then may pass into the blood, disseminate into different tissues, and migrate into the testes through a barrier similar to the blood-brain barrier. Thus, predicting the efficiency of transmission from donor blood to recipient blood may not relate directly to the actual transmission event, i.e., the virus population moving through various genetic bottlenecks and barriers (5). Different barriers during this transmission process may place the inoculating HIV-1 population under different, possibly distinct selective pressures leading to systemic infection by one predominant HIV-1 clone. We recently provided *in vivo* evidence that an HIV-1 population and not a single clone infects the vaginal tract during male-to-female transmission and that a possible genetic bottleneck from the vaginal tract to the blood results in systemic infection by a single HIV-1 clone (6). However, it is still not fully understood if this transmission bottleneck is a stochastic event or involves selection for a specific viral function/activity which may be mapped to viral protein structure(s), linear sequence(s), or posttranslational modifications. It is also unclear how genital microbiome, innate immune status, and other host factors could influence this HIV clonal selection during transmission (7–10). For the development of a successful preventive HIV-1 vaccine, it will be critical to obtain in-depth knowledge of the genotypic and phenotypic properties of the transmitted compared to the nontransmitted HIV-1 clones.

When comparing blood-derived HIV-1 from chronic and acute infections, we and others observed no differences in receptor usage, sensitivity to entry inhibitors, and cell tropism (11, 12). Interestingly, the chronic HIV-1, tested as a virus population and not a single clone, appears to have higher replicative fitness and greater host cell entry efficiency than the acute HIV-1, typically a single clone (12, 13). However, many studies have focused on identifying signature sequences of the TF clones, making it distinct in the inoculating HIV-1 swarm from the human donor (14–16). For example, alpha interferon (IFN-α) resistance encoded by the *gag* gene may be a distinct feature of TF HIV-1, but this has been the subject of debate (9, 11, 17). Typically, TF viruses have been shown to utilize CD4 and CCR5 for cell entry (3). Additionally, fewer potential N-linked glycosylation sites (PNGS) and shorter variable loops have been linked to the TF phenotype (18). One study showed that infectious molecular clones of TF were more infectious and expressed more envelope on the surface than HIV-1 molecular clones derived from chronic disease (17). However, there is still considerable debate about Env properties of TF HIV-1. A reduced glycan shield on TF HIV-1 (9, 19, 20) was thought to improve replicative fitness and/or host cell entry; i.e., it may be a favorable trait for transmission. However, TF clones with fewer N-linked glycan sites had no clear advantage in terms of replicative rate, receptor/coreceptor binding, or entry efficiency over HIV-1 found during chronic disease (11, 12, 21). The factors governing infection by HIV-1 harboring Env with fewer N-linked sites during acute infection remain poorly understood. Clearly, the emergence of HIV-1-specific antibodies following early infection selects for new N-linked sites, especially in the Env hypervariable loops, which corresponds to viral escape from this immune pressure (22). Compared to chronic HIV-1, TF viruses may bind more efficiently to dendritic cells (DCs) for transmission in *trans* to CD4^+^ T cells (17, 23), but other studies suggest the opposite (12, 14).

The impact of the Env on the replication efficiency of HIV-1 isolates in primary T cells, macrophages, and DCs has been well described (12, 21), but the role of Env in transmission is poorly understood. Thus, the HIV-1 *env* genes from individuals during acute/early and chronic disease were analyzed in the context of the same NL4-3 HIV-1 backbone. Acute (Env_acute_) and chronic (Env_chronic_) Env chimeric viruses were then tested in multivirus competition assays in human *ex vivo* penile and cervical tissue to determine if specific Env properties influence the transmission fitness. Differential transmission fitness was compared to replicative fitness in primary CD4^+^ T cells and C-type lectin binding affinity. C-type lectin receptors are a family of transmembrane and soluble receptors containing carbohydrate recognition domains (24), expressed mostly by myeloid cells. They play critical roles in both innate and adaptive immunity and have been shown to have important protective functioning at mucosal barriers while also facilitating binding and capture of HIV-1 by antigen-presenting cells (APCs) (25). Here, transmission fitness experiments were carried out in penile and cervical tissue following incubation with mannan, a high-mannose polysaccharide, to saturate C-type lectins, found throughout the mucosal tissues by immunofluorescent staining. Based on the studies described herein, increased transmission fitness of HIV-1 may relate to “breaking” the C-type lectin trap in mucosal tissue.

## RESULTS

### Multivirus infection in human explant tissue as a model for mucosal HIV-1 transmission fitness.

**FIG 1:**
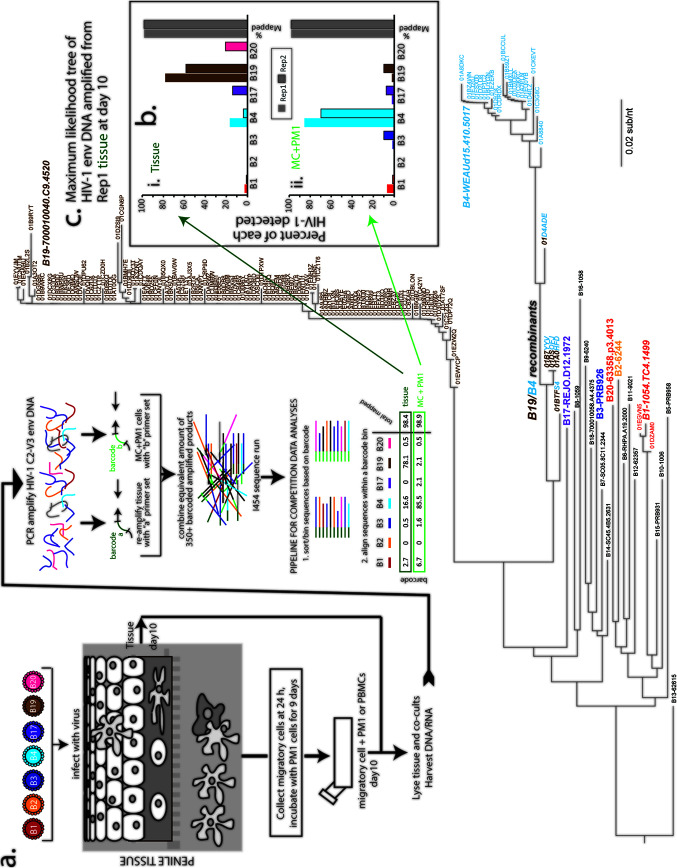
Schematic of transmission fitness assay and analyses of multivirus infections. (a) Human explant tissue was exposed to a mixture of Env_acute_ and Env_chronic_ viruses for 3 h, washed, and cultured overnight. Cells migrating out of the tissue were collected and cocultured with PM1 CD4^+^ T cells. After10 days, both tissue and MC+PM1 cocultures were lysed, and DNA was extracted for PCR amplification of the C2-V3 envelope region. After ligation of barcoded oligonucleotides, purification, and quantification, equivalent amounts of products were subject to NGS. Data analysis was performed using SeekDeep, a custom pipeline sorting sequences based on barcodes and aligning sequences within barcode bins. (b) Results of multivirus infections are presented as percent sequence reads of each virus detected in the assay in tissue or MC+PM1 cocultures. Gray bars indicate the percentage of sequences from that barcoded PCR product (representing this multivirus competition) mapped to one of the seven viruses in the competitions. It is important to note that these analyses involve counting HIV isolates based on phylogenetic clustering, where low-frequency mutations do not impact the analyses. This was verified as shown in panel c. The relative amount of each virus in the competition was determined using maximum-likelihood trees of the HIV-1 C2-V3 *env* DNA amplified from tissue or MC+PM1 cocultures. Env sequence in the maximum likelihood tree of panel c in the bold and larger colored text and the black text represent the C3-V3 gp120 sequences of all the chimeric Env HIV-1 strains that were employed in the various multi-virus competitions used in this study (see below). For the representative multi-virus competition results shown in this figure, the Env chimeric viruses used in this competition are the branch points on the tree labeled with the colored text. The small text labels (text not important) show the individual branches/sequences defined as NGS reads "counted" from the multivirus competitions by SeekDeep. The reference sequences for the viruses in this competition are shown in italic, bold, large text, namely B1-1054.TC4.1499 (red), B2-6244 (yellow), B3-PRB926 (dark blue), B4-WEAUd15.410.5017 (light blue), B17-REJO.D12.1972 (purple), BB19-700010040.C9.4520 (brown), and B20-63358.p3.4013 (orange on the tree but pink for rest of figure). A few recombinant B19/B4 sequences were identified in this competition and were identified as a mixed brown/light blue text label. Of all HIV sequences in the maximum-likelihood tree (c) from the matched-input viruses (B1, B2, B3, B4, B19, and B20), only B1, B4, and B19 replicated in the tissue (see replicate 1 of the tissue in panel b). Less than 1%, or 4 sequences, were recombinants of B4 and B19 with different breakpoints.

To approximate transmission fitness, we employed an *ex vivo* tissue model to test the transmissibility of both acute and chronic Env chimeric viruses. Eleven Env_acute_ and three Env_chronic_ viruses that were previously constructed and characterized (12, 26) were used in this study. Phylogenetic analysis and characteristics of all Env_acute_ and Env_chronic_ viruses employed in this study are presented in the phylogenetic tree in Fig. 1c. Titers of all viruses were determined on phytohemagglutinin (PHA)/interleukin 2 (IL-2)-treated peripheral blood mononuclear cells (PBMCs) using the standard TCID_50_ (50% tissue culture infective dose) method. In the example illustrated in Fig. 1, human penile glans tissue was exposed to a mix of seven Env_acute_ HIV-1 (B1, B2, B3, B4, B17, B19, and B20), each at 150 infectious units (IU), for 3 h and then washed and cultured overnight. At 24 h, migratory cells (MC) were collected and cocultured with PM1 clonal CD4^+^ T cells. Following 10 days in culture, tissue and MC+PM1 cocultures from the mixed infections were analyzed for relative virus production.

As detailed in Materials and Methods, barcoded Env C2-V3 amplicons were sequenced from tissue and MC+PM1 cocultures from each mixed-virus competition. SeekDeep (27) was then used to count sequence reads aligning to each virus added to the competition (Fig. 1a). The relative percentages of the B1, B2, B3, B4, B17, B19, and B20 viruses obtained are shown in Fig. 1b. Additionally, C2-V3 sequences from the same barcoded product were aligned to Env_acute_, and the maximum-likelihood tree in Fig. 1c shows one representative phylogenetic tree of the C2-V3 sequences amplified from the tissue at 10 days after multivirus infection. As described above, the Env consensus sequence ID labels of viruses used in this study is shown in Fig. 1c (in black text), while the virus ID labels specific for this mixed-virus competition are shown in colored text (Fig. 1c). The maximum-likelihood tree (Fig. 1c) and the SeekDeep analyses (Fig. 1b) were congruent and show that Env_acute_ B19 (nearly 80%) followed by B4 dominated the multivirus competition in tissue, whereas B1 was found at a low percentage. The <1% not mapping to one of the seven HIV-1 isolates were identified as recombinants of B4 and B19 (Fig. 1c).

Despite the high level of B19 replication in the tissue, this virus was not identified in the MC+PM1 cocultures, suggesting that this virus was not carried by the MC out of the tissue. In contrast, the B4 virus was efficiently carried through the tissue to infect and propagate in CD4^+^ T cells (Fig. 1b). In the subsequent multivirus competitions, we found low levels of stochastic infection by all Env_acute_ and Env_chronic_ HIV-1 of the tissue and transmission to T cells. However, in general, Env_acute_ viruses were more readily transmitted through the penile and cervical tissue to infect T cells, whereas other HIV-1 Env strains (i.e., Env_chronic_) propagated primarily in the tissue.

**FIG 2:**
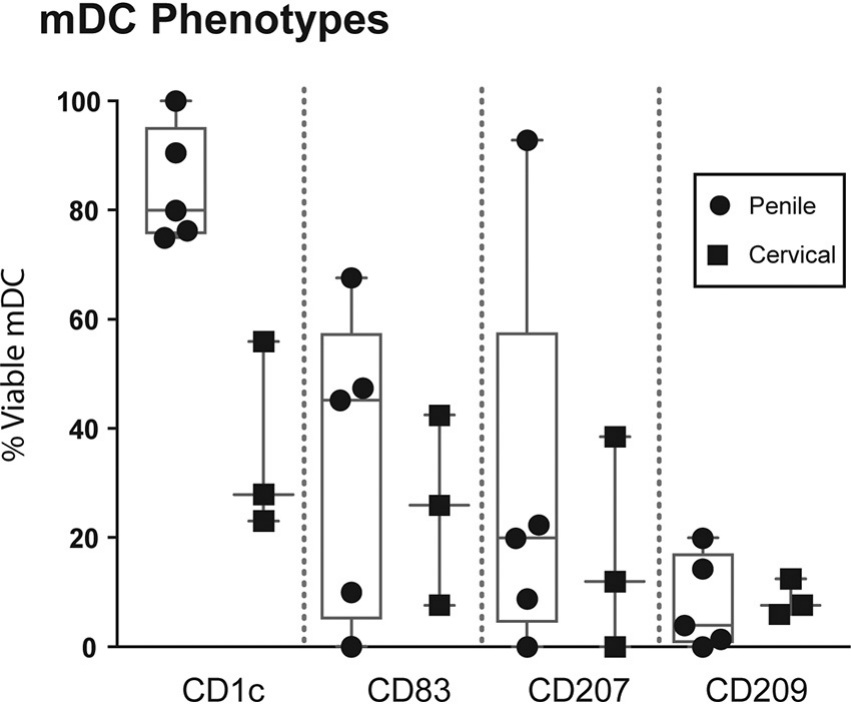
Characterization of the migratory cell phenotype in penile and cervical tissue. The percentages of CD1c-, CD83-, CD207-, and CD209-positive cells of all viable mDCs isolated from penile (*n* = 5) and cervical (*n* = 3) tissue were assessed by a multicolor flow panel on an LSRIIFortessa flow cytometer. Box-and-whisker plots show median, minimum/maximum, and 25th/75th percentiles of the percentage of viable cells stratified by CD1c, CD83, CD207, and CD209 expression. Each symbol represents an individual penile or cervical tissue donor.

To phenotypically characterize the emigrating DCs, tissue blocks were cultured overnight and MC were collected and stained with a multicolor antibody cocktail for flow cytometry. Analysis of the phenotype present within the mDC population derived from penile (*n* = 5) and cervical (*n* = 3) tissue revealed that 80% and 28% are CD1c positive, 20% and 12% are CD207 (langerin) positive, and 3.95% and 7.69% are CD209 (DC-SIGN) positive (Fig. 2), which is in accordance with previous findings (28–30).

### Higher transmission fitness of acute over chronic Env chimeric viruses in the human penile and cervical tissue transmission model.

**FIG 3:**
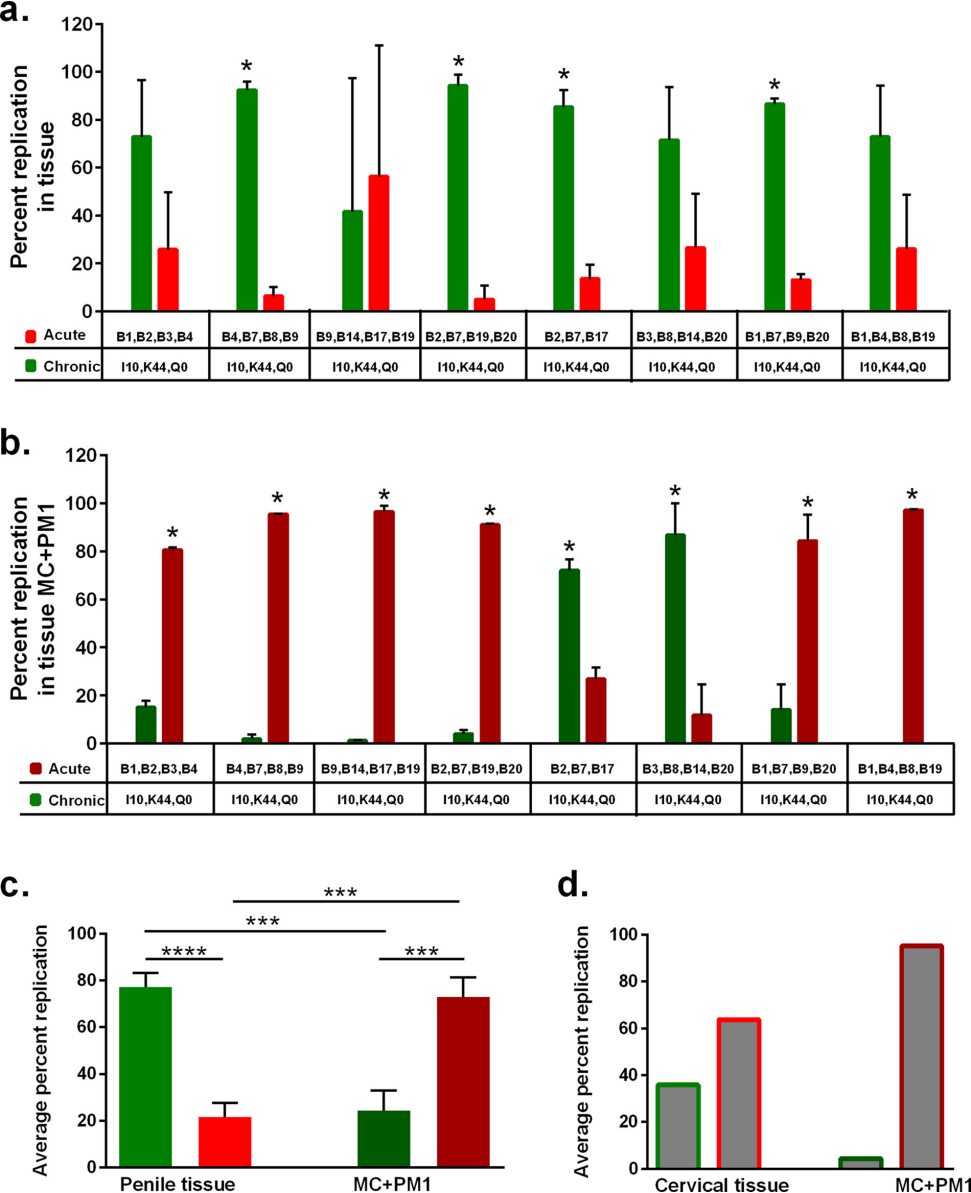
Higher transmission fitness of Env_acute_ versus Env_chronic_ viruses in multivirus competition assays in human tissue. (a) Penile tissues were dissected into 3-mm^3^ explants and exposed to eight different mixtures of a panel of Env_acute_ viruses and the same three Env_chronic_ viruses. (b) Following overnight culture, migrating cells from the tissue were collected and cocultured with PM1 T cells (MC+PM1). After 10 days in culture, DNA from tissue and MC+PM1 cells was isolated, PCR amplified, and analyzed by NGS. The cumulative percent replication in penile tissue (a) and in MC+PM1 cocultures (b) of Env_chronic_ and Env_acute_ viruses for tissue A and tissue B in each of the eight multivirus panels is shown. (c) The combined average replication of all Env_chronic_ and all Env_acute_ viruses of the eight virus competitions in penile tissues and Env_chronic_ (green bars) and Env_acute_ (red bars) viruses in MC+PM1 cocultures was determined. Each competition was done in triplicates in tissues from two donors. (d) Average replication of Env_chronic_ (bars with green outlines) and Env_acute_ (bars with red outlines) viruses of one competition set in cervical tissue and the corresponding MC+PM1 cocultures were evaluated by NGS. Statistical significance was determined using the Holm-Sidak method (a and b) (*, *P* < 0.05) and a two-tailed Mann-Whitney test (c and d) (***, *P* < 0.001; ****, *P* < 0.0001).

Due to limited availability of cervical tissue, multivirus competitions were predominantly performed in human penile tissue as a model for transmission fitness. Tissue was exposed to a mix of three or four Env_acute_ viruses (B1, B2, B3, B4, B7, B8, B9, B14, B17, B19, and B20) plus three Env_chronic_ viruses (I10, K44, and Q0), each virus at 150 IU. Eight different mixtures of Env_acute_ viruses ensured that all 11 Env_acute_ viruses competed against each other and with the same three Env_chronic_ viruses (Fig. 3). The relative production of each virus in tissue and MC+PM1 multivirus competitions was analyzed as described in Fig. 1. Relative replication of the total Env_acute_ versus Env_chronic_ viruses was compared in tissue and MC+PM1 cocultures (Fig. 3a and b). With the exception of one mixed-virus competition (B9, B14, B17, B19, I10, K44, and Q0), Env_chronic_ outreplicated Env_acute_ in penile tissue (Fig. 3a), to a significant degree in 4 mixed-virus competitions. Even though the chronic virus represents only 43 to 50% of the inoculating virus, chronic virus represented 80% of the virus replicating in the tissue (7 of 8 competitions) (Fig. 3a and c).

In complete contrast to the preferential Env_chronic_ replication in the tissue, Env_acute_ significantly outreplicated Env_chronic_ HIV in the MC+PM1 cocultures in 6 of the 8 multivirus infections (Fig. 3b and c). For this to occur, Env_acute_ HIV-1 must have been preferentially carried out of the tissue by MC and then transferred to PM1 cells. Combined, Env_acute_ HIV-1 contributed to over 80% of the virus replication in all but two multivirus infections (Fig. 3b and c). In these two multivirus infections (B2, B7, B17, I10, K44, and Q0 and B3, B8, B14, B20, I10, K44, and Q0), Env_chronic_ not only was transmitted to the T cells but also replicated within the tissue (Fig. 3a and b).

**FIG 4:**
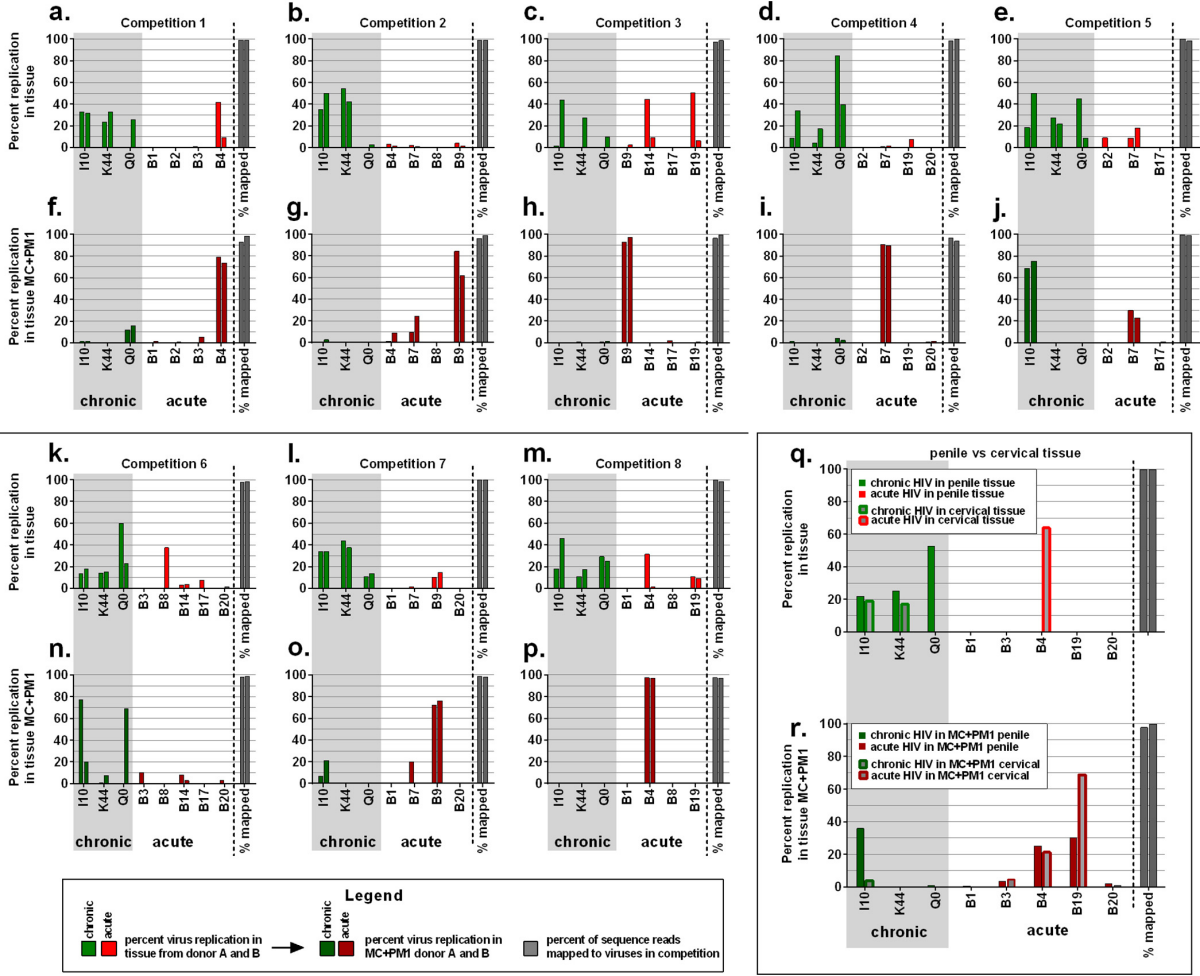
Multivirus competition assays to assess the transmission fitness across penile and cervical tissue. Penile and cervical explants were exposed to mixtures of three Env_chronic_ viruses (I10, K44, and Q0) and three to five Env_acute_ viruses (B1, B2, B3, B4, B7, B8, B9, B14, B17, B19, and B20) for 3 h. After overnight culture, migrating cells from the tissue were collected and cocultured with CD4^+^ PM1 T cells (MC+PM1). Following 10 days in culture, both tissue and MC+PM1 cocultures were lysed and PCR amplified, and replication of viruses was analyzed by NGS. (a to p) Percent replication of eight different mixtures of Env_chronic_ and Env_acute_ viruses in tissues from donor A and donor B and Env_chronic_ and Env_acute_ viruses in the corresponding MC+PM1 cocultures. Each graph shows the percentage of sequence reads for donor A and donor B from the NGS analysis mapped to the viruses present in the competitions. (q and r) Side-by-side comparison of Env_chronic_ and Env_acute_ virus replication in penile tissue and Env_chronic_ and Env_acute_ virus replication in cervical tissue (q) and comparison of transmission fitness of Env_chronic_ and Env_acute_ viruses in MC+PM1 derived from penile tissue with transmission fitness of Env_chronic_ and Env_acute_ viruses in MC+PM1 from cervical tissue (r) of one competition set (I10, K44, and Q0 and B1, B3, B4, B19, and B20).

Analyses of individual virus replication in the competitions (Fig. 4), differential transmission fitness of acute HIV-1 was evident in 8 different multivirus infections in penile tissue. Of the 11 Env_acute_ viruses, B4, B7, and B9 consistently showed the highest transmission fitness when combined with the same three Env_chronic_ viruses but different combinations of the other 8 Env_acute_ HIV-1 (Fig. 4f, g, h, i, j, o, and p). When B4, B7, and B9 (Fig. 4b and g) or B7 and B9 (Fig. 4l and o), were in competition together, the acute B9 virus was preferentially transmitted through the tissue by MC to replicate in PM1 cells. In the two competitions where Env_chronic_ was transmitted more efficiently than Env_acute_ (Fig. 4e, j, k, and l), the highly “transmissible” B4 and B9 were not included in the multivirus competitions.

### Comparing HIV-1 transmission fitness in an endocervical versus penile glans tissue model.

Penile and endocervical tissues were exposed to I10, K44, QO, B3, B4, B19, and B20 using the experimental procedures described above (Fig. 1). The relative replication in the penile and cervical tissue was similar in that Env_acute_ was again preferentially transmitted through the tissue to infect T cells via MC (Fig. 4q and r). We frequently observed stochastic replication of Env_acute_ in the tissue along with Env_chronic_ in these multivirus competitions. For example, high-level B4 replication was observed in multivirus competitions in the penile tissue (Fig. 4a and m) as well as in the endocervical tissue (Fig. 4q). In contrast, when Env_acute_ viruses with high transmission fitness were present in the multivirus competitions, Env_chronic_ HIV-1 were not transmitted and were almost completely absent in the MC+PM1 cocultures (Fig. 4f, g, h, i, o, p, and r). The patterns of relative HIV-1 replication were similar in experiments using penile and cervical tissue, i.e., Env_chronic_ viruses had sequestered replication in the tissue, while Env_acute_ viruses are preferentially taken up by MC and transferred to T cells (Fig. 3d). Replication of Env_acute_ B4 in the cervical tissue from one of the mixed-virus competitions in one of two donors highlights the stochastic replication of HIV-1 in tissue with some mixed-virus competitions. Nonetheless, Env_acute_ HIV-1 viruses were still preferentially transmitted by the migratory cells from the cervical tissue to free CD4^+^ T cells (Fig. 3d).

### Transmission fitness does not correlate with replicative fitness in peripheral blood mononuclear cells.

**FIG 5:**
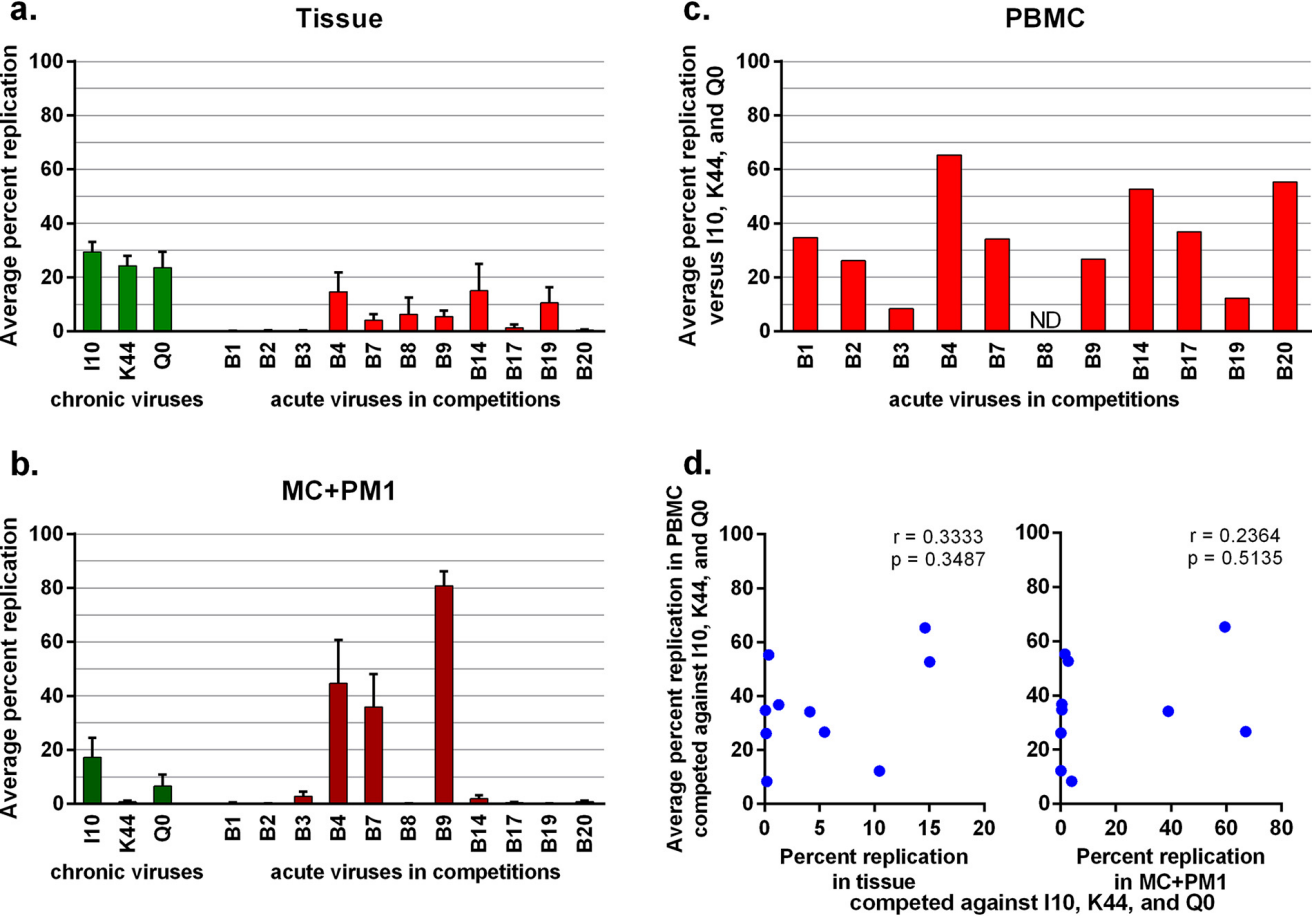
Replication fitness does not predict transmission fitness. Multivirus infection assays in penile tissue were performed, and the average replication of each Env_chronic_ and each Env_acute_ virus in tissue (a) and in MC+PM1 cocultures (b) was determined by sequencing. (c) Each of the Env_acute_ viruses was competed against the Env_chronic_ controls in PHA- and IL-2-activated PBMCs to assess the pathogenic fitness (21). The average pathogenic fitness versus the three Env_chronic_ viruses is shown. (d) The percent replication of Env_acute_ viruses in competitions in tissue and in MC + PM1 cocultures was plotted against the average percent replication in PBMC competitions. ND, not done.

The average relative fitness of each virus in the tissue or transmitted by MC to the T cells is shown in Fig. 5a and b (respectively) from the total of all 8 multivirus competitions performed in penile tissues from two donors. Again, B4, B7, and B9 had the highest transmission fitness using this penile explant model. We had previously reported on the replicative fitness of each acute virus in competition against the same three Env_chronic_ viruses (I10, K44, and Q0) in PHA- and IL-2-treated PBMCs (21, 31) (Fig. 5c). Dual-virus competitions in PBMCs showed that all Env_acute_ viruses could replicate with lower or higher fitness than the competing Env_chronic_ viruses (Fig. 5c). Replication of B1, B2, B3, B17, and B20 was low or undetected in both tissue and MC+PM1 cocultures in the penile tissue model (Fig. 5a and b), whereas the same stocks replicated efficiently in PBMCs (Fig. 5c). In Fig. 5d, the average replicative fitness of the Env_acute_ viruses in PBMCs (from Fig. 5c) is plotted against the replication of these viruses in tissue and MC+PM1 cocultures derived from the multivirus competitions in penile tissue (from Fig. 5a and b). Previous studies revealed a direct correlation between the replicative fitness of the infecting HIV-1 (as determined in PBMCs and primary T cells) and subsequent disease progression (26, 31–33). We observed no correlation between replicative fitness of Env_acute_ HIV-1 with the replicative fitness in tissue or with the ability of the virus to be transmitted by MC to replicate in T cells (i.e., transmission fitness) (Fig. 5d).

Collectively, these data suggest that replication fitness as measured in PBMCs is different from the transmission fitness assessed in the *ex vivo* human penile explant model. As discussed below, replicative fitness of HIV-1 in free T cells was unlikely to predict the efficiency of viruses to penetrate the mucosa and be bound by MC to trans-infect T cells, which we refer to as transmission fitness.

### Transmission fitness is linked to C-type lectin binding affinity.

**FIG 6:**
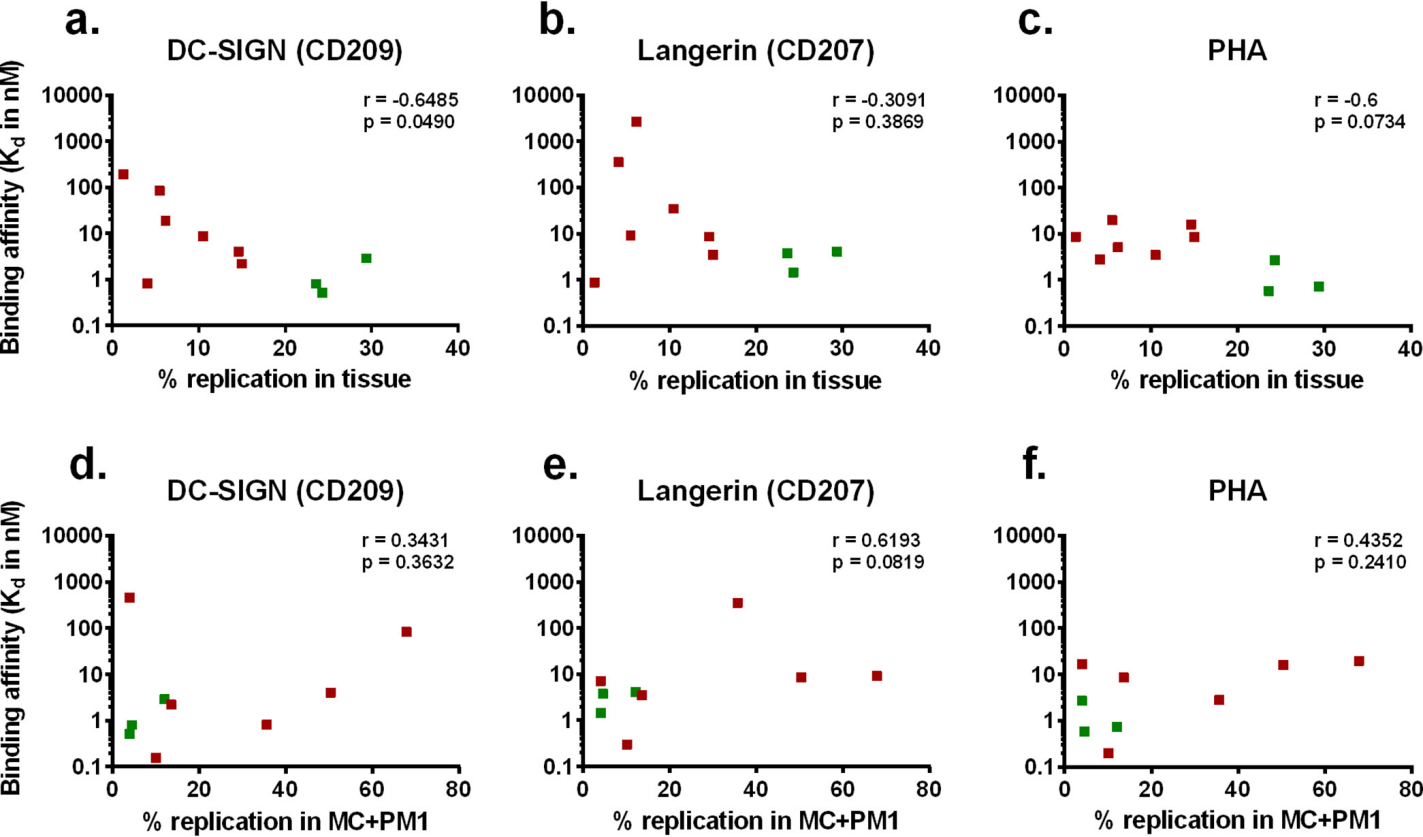
Binding profile of Env_acute_ and Env_chronic_ viruses to C-type lectins. Binding to DC-SIGN and langerin was determined using an acoustic biosensor. Binding affinity (dissociation constant [*K_d_*], in nanomolar units) was determined by binding the lectins of interest to the chip and allowing viruses to flow over the surface. Average replication in tissue of Env_acute_ (red squares) and Env_chronic_ (green squares) viruses was correlated with the binding affinity to DC-SIGN (a), langerin (b), and PHA (c). Average transmission fitness in MC+PM1 cocultures of Env_acute_ (red squares) and Env_chronic_ (green squares) viruses was plotted against the binding affinity to DC-SIGN (d), langerin (e), and PHA (f). PHA was tested as the nonspecific control. Each symbol represents one virus. Statistical analysis was performed using a Spearman rank correlation analysis.

Based on previous studies, these analyses of Env_acute_ versus Env_chronic_ could not define a specific amino acid sequence, altered function, or phenotype (12, 13). The only unique feature appeared to be fewer conserved N-linked sites for some of the Env_acute_ HIV-1 than Env_chronic_ HIV-1 viruses. Based on these observations, we proposed that the Env glycoproteins of acute HIV-1 may have differential binding affinity to C-type lectins. Binding affinities of Env_acute_ and Env_chronic_ viruses for DC-SIGN and langerin were measured on a RapID4 acoustic biosensor. In this system, DC-SIGN and langerin were covalently bound to the surface of a quartz crystal chip. Inactivated Env_acute_ and Env_chronic_ virus stocks were passed over the chip surface, and kinetic binding parameters were measured by the variation in resonant frequency. The binding affinity of each virus for DC-SIGN, langerin, and PHA was then compared to the replication efficiency in both tissue and MC+PM1 cocultures (Fig. 6). The ability of the viruses to replicate in the tissue was related to DC-SIGN binding affinity (Fig. 6a). There was also a trend for viruses that replicated in tissue to bind with higher affinity to langerin (Fig. 6b). While we observed a 1,000-fold variation in langerin and DC-SIGN binding affinity, all viruses had similar binding affinity to PHA (<50-fold variation in dissociation constants) (Fig. 6c). There were, however, no correlations between binding affinity to these C-type lectins and transmission fitness, i.e., the ability to bind MC, pass through the tissue, and infect free CD4^+^ T cells (Fig. 6d to f).

### C-type lectins trap HIV-1 replication in the tissue.

**FIG 7:**
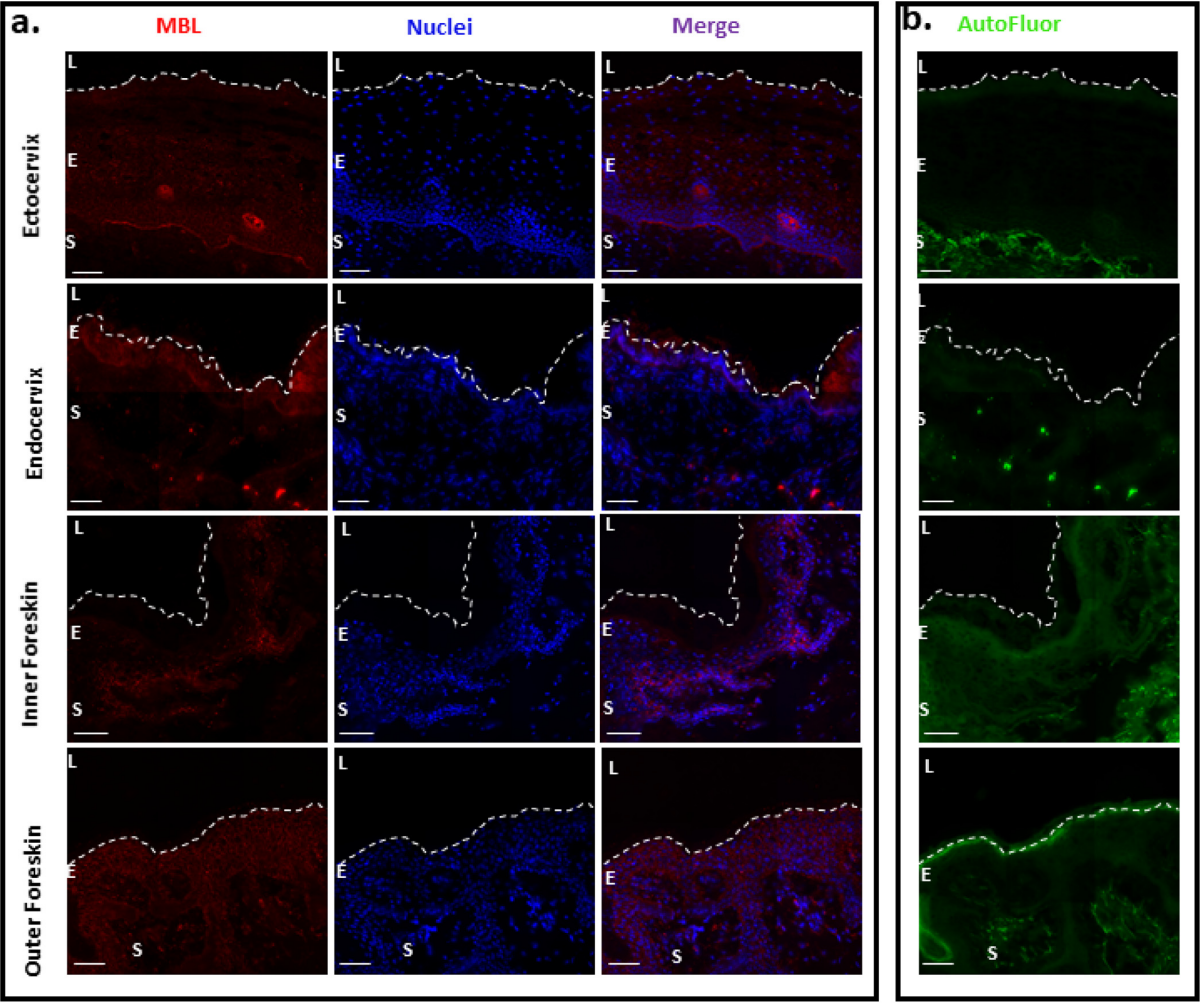
Presence of MBL in different mucosal tissue types. (a) Fluorescent deconvolution microscopy images of ectocervix, endocervix, inner foreskin, and outer foreskin stained for MBL (red) and DAPI (blue). (b) Autofluorescence (green) of the corresponding tissues. Magnification, ×40. Bar, 40 μm. Dashed lines indicate the luminal surface. L, lumen; E, epithelium; S, stroma.

Previous studies have described high levels of soluble C-type lectins, like mannose-binding lectins (MBLs), in mucosal tissue as well as Dectin-1, a C-type lectin on epithelial and endothelial cells. MBLs are thought to trap pathogens with high-mannose glycans in the mucosa and prevent translocation (34). Our fluorescent deconvolution microscopy confirms the presence of MBL in different mucosal tissues (Fig. 7). These analyses serve only to illustrate that DC-SIGN and langerin on the myeloid cell lineage in mucosal tissue provide relatively few binding sites for high-mannose glycans compared to the abundant MBL in these same tissues. Previous studies have shown that the HIV-1 Env gp120/gp41 found on the virus surface retains N-linked high-mannose glycans and somehow avoids late Golgi complex (GC) modification responsible for trimming and complex glycan content (35, 36).

**FIG 8:**
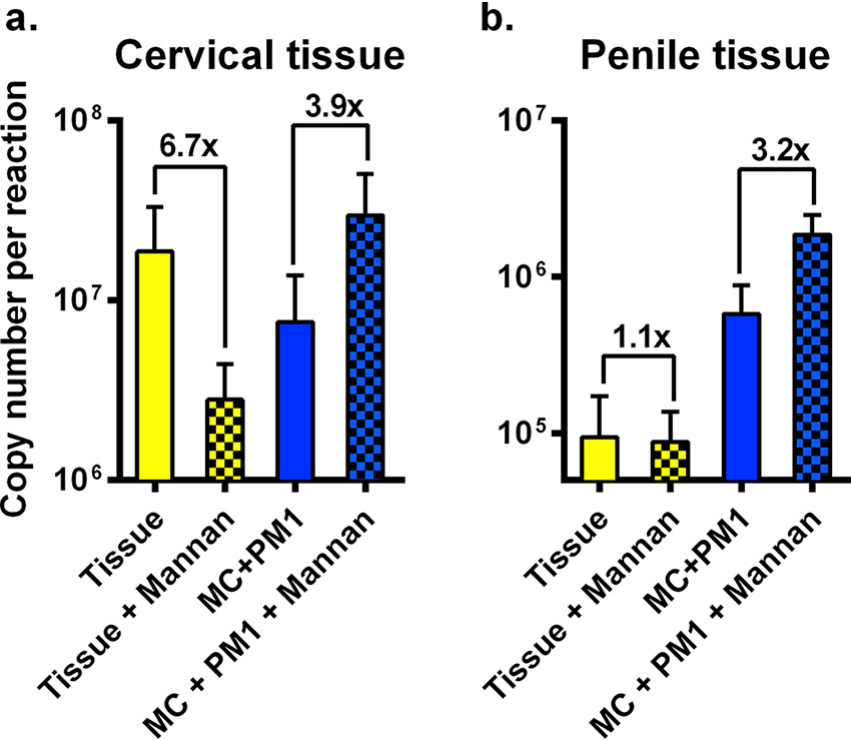
Mannan alters the transmission of HIV-1 across mucosal tissue. (a) Cervical and (b) penile tissues were incubated in medium alone or medium containing mannan for 12 h before exposure to a mix of one Env_acute_ and one Env_chronic_ virus. Migratory cells were harvested after 24 h and cocultured with PM1 T cells. After 10 days in culture, the total HIV-1 copy number in tissue and MC+PM1 cells with and without mannan was assessed using a *gag*-specific qPCR. Each assay was performed with tissue from three donors. The values are means and standard errors of the means.

To evaluate the influence of glycans on the transmission fitness, penile and cervical explants were pretreated with mannan for 12 h and washed prior to addition of one acute B4 and one chronic Q0 virus. Saccharomyces cerevisiae mannan, derived from N linkage, has a conserved mannose(×8)–*N*-acetylglucosamine(×2) core structure with high affinity to all C-type lectins. Replication of virus in both tissue and MC+PM1 was evaluated by a Gag-specific quantitative PCR (qPCR). Analysis of the total viral copy number revealed that cervical tissue soaked in mannan prior to virus exposure exhibited 6.7× reduced viral replication compared to untreated tissue (Fig. 8a). In penile tissue, no differences in viral replication were observed between mannan-treated and untreated tissue (Fig. 8b). In contrast, 3.2× and 3.9× increased HIV-1 copy numbers were found in MC+PM1 cocultures derived from penile and cervical tissue, respectively, when tissue was treated with mannan (Fig. 8a and b). These findings suggest that fewer viruses were trapped in tissue treated with mannan and that more viruses were able to cross the mucosa with MC to infect T cells.

## DISCUSSION

In the vast majority of new HIV-1 infections, a stringent transmission bottleneck leads to the establishment of a systemic infection by a single viral founder (incidence estimated to be between 76 and 90%) (3). A recent study suggests that this dramatic decline in the HIV-1 heterogeneity from donor to recipient during transmission may be related to a genetic bottleneck during the virus inoculation in the recipient as well as during the translocation of the HIV-1 from the recipient genital mucosa to the blood (6). It remains poorly understood if the bottleneck leading to systemic infection by a single HIV-1 variant is caused by a stochastic event or if selective factors influence the efficiency of HIV-1 transmission through the genital mucosa. During transmission, the HIV-1 genetic bottleneck and any selection (as opposed to stochastic effects) would likely have multifactorial origins. For example, the genetic bottleneck maybe the result of a lower activation state of CD4 T cells (37) and/or the fact that in any transmission event, the majority (>93%) of HIV proviruses are defective (38).

In this study, we evaluated the transmission fitness of HIV-1 harboring subtype B chronic and acute Env glycoproteins by employing a multivirus competition infection of primary human penile and cervical tissue. Env chimeric clones were chosen to specifically determine the role different Env phenotypes play in the mucosal transmission event. In previous studies, replicative fitness in primary CD4^+^ T cells and macrophages was dominated by the efficiency of host cell entry and mapped to the *env* gene (39, 40). By using chimeric clones that share the same backbone, we limited transmission efficiency to Env derived from HIV-infected patients during acute or chronic infection. Although Env activity may be dominant or an important trait for transmission, we are not discounting other HIV-1 proteins/factors that may impact transmission efficiency (9, 11, 17). Our data show that Env_chronic_ viruses replicate mainly in the mucosal tissue, while Env_acute_ viruses pass through the tissue with migratory cells and initiate infection in CD4^+^ T cell lines. Replication of HIV-1 trapped in tissue directly correlated with binding affinity to C-type lectins. Treating the tissue with a saturating concentration of mannan resulted in higher efficiency of HIV-1 transmission to T cells.

Based on our tissue model, HIV-1 harboring Env with the highest binding affinity to C-type lectins showed increased virus replication in the mucosal tissue but was not efficiently transmitted through to CD4^+^ T cells. Our studies suggest that C-type lectins may “trap” HIV-1 replication in the mucosa and even prevent translocation for systemic infection. Mucosal biologists/immunologists have known for decades that C-type lectins are highly abundant in foreskin, penile, vaginal, and cervical tissues (41, 42) and are key innate barriers preventing pathogen translocation. Understanding the role of C-type lectins in HIV-1 transmission has a long and convoluted history. As early as 1997, Garred et al. (43) described protection from HIV-1 in high-risk cohorts with a specific promoter polymorphism in mannose-binding lectin loci which led to reduced expression of mannose-binding lectin (MBL). However, the protective role of C-type lectins in HIV-1 infection was soon eclipsed by studies describing a role of DC-SIGN in capturing and transmitting HIV-1 (44), which remains a controversial topic (45, 46). Langerhans cells (LCs) expressing langerin or DCs expressing DC-SIGN are probably the first immune cells to come into contact with infecting virus in these mucosal tissues. The proposed role of LCs in HIV-1 transmission was questioned in a study describing the capture, endocytosis, and degradation of HIV-1 by Birkbeck granules following binding to langerin on mucosal LCs (47).

Our findings suggest that replicating HIV-1 in the genital mucosa may be trapped by soluble MBL, shown to have binding specificity for mannose similar to that of the less abundant DC-SIGN and langerin found on DCs and LCs, respectively (48, 49). Addition of mannan to genital mucosal tissue would saturate C-type lectin binding and as a result, reduce trapped HIV-1 replication in the tissue. Surprisingly, addition of mannan increased HIV-1 transmission through the tissue to infect T cells via tissue migratory cells. These findings suggest competing roles of C-type lectins in mucosal tissue to prevent HIV-1 translocation for systemic infection versus the role of DC-SIGN and langerin in enhancing heterosexual HIV-1 transmission. Again, C-type lectins in mucosal tissue are a primary innate defense mechanism preventing pathogen translocation (50, 51), but this process has not been well defined for HIV-1. There is of course the added complication that unlike pathogenic microbes that harbor surface glycoproteins with high-mannose glycans (e.g., *Chlamydia* and Candida albicans), HIV-1 glycoproteins are subject to the same posttranslational machinery as cellular glycoproteins, typically resulting in greater glycan diversity and reduced branching. However, Doores et al. (36) described high-mannose glycans on Env directly from virus particles, whereas recombinant Env, produced from the same cell types, had more complex glycans, similar to that observed on cellular glycoproteins. These findings suggest that HIV-1 Env may escape the medial or trans GC processing in the context of virus replication, possibly through the coexpression of HIV-1 Nef or Vpu accessory proteins.

In summary, we determined that Env_acute_ HIV-1 viruses were more readily transmitted in penile or cervical tissue than Env_chronic_ viruses. Trapped replication in these mucosal tissues was observed with Env_chronic_ viruses and was associated with higher C-type lectin binding affinity. As described herein and as previously reported, the soluble MBL is omnipresent in genital mucosal tissues and serves as an innate defense mechanism to prevent microbial translocation. We propose that HIV-1 particles with high mannose content may be retained in the mucosa and that HIV-1 with Env with reduced glycan/mannose content may break this trap for systemic infection. These findings are consistent with our recent observation that a more diverse HIV-1 population infected the female genital tract while a single HIV-1 variant was found in the blood at early infection (6). Future studies will focus on determining the actual glycan composition on the Env glycoprotein of the transmitter/founder HIV-1 clone that is responsible for new infections and avoids the lectin trap in genital mucosa.

## MATERIALS AND METHODS

### Acute and chronic Env chimeric viruses.

Env_acute_ and Env_chronic_ were constructed from 11 acute subtype B envelopes obtained from the Center for HIV-1/AIDS Vaccine Immunology (CHAVI) acute infection studies (3) and three chronic envelopes from patient samples from a Belgian cohort (12, 26). Env chimeric viruses were constructed using a yeast-based recombination cloning method as previously described (52). Briefly, the envelope genes from acute and chronic clones were recombined into pREC_nfl_NL4-3_Δenv/URA3 following transfection into Saccharomyces cerevisiae. Virus was produced by transfection of the pREC construct with the supplementary vector pCMV_cplt into 293T cells using the Effectene lipid system (Qiagen, USA) according to the manufacturer’s instructions. All chimeric viruses were tested for coreceptor usage on U87.CD4 cells expressing either CCR5 or CXCR4 (12, 21) (all were CCR5 using) and then propagated, and titers were determined on U87.CD4.CCR5 cells using the 50% tissue culture infective dose (TCID_50_) assay. Subsequent experiments on transmission fitness involved tissue explants that harbor a variety of lymphoid and myeloid cells that are susceptible to HIV-1 infection. Previous studies have shown that these HIV-1 stocks differ by 1 log in absolute TCID_50_ titers derived from PBMCs and primary T cells from different donors and from T cell lines, but the relative difference in titers remained similar. The acute viruses are termed B1, B2, B3, B4, B7, B8, B9, B14, B17, B19, and B20, and the three chronic control Env chimeric viruses are named I10, K44, and Q0 (12). Neighbor-joining phylogenetic tree analysis was performed to compare sequences of the original acute HIV-1 *env* genes derived from single genome amplification (SGA) to those of the acute chimeric *env* viruses and to highlight the diversity within the chronic *env* chimeric viruses. Neighbor-joining trees were constructed with SEAVIEW 4 and visualized with FigTree 1.4.2.

### Ethics statement.

Human penile and cervical tissue was collected with written consent according to local research committee (LRC) guidelines. All tissues were obtained under protocols approved by the Imperial College NHS Trust Tissue Bank and the National Research Ethics Committee in accordance with the Human Tissue Act 2004.

### HIV-1 transmission fitness assay in human explant tissue.

For this study, for lack of a better term, we describe *ex vivo* transmission fitness as the ability of the virus to enter the tissue, to be carried by and/or infect migratory cells, and then to be transferred to free T cells.

Deidentified penile tissue was obtained from men undergoing gender reassignment surgery at Charing Cross Hospital, London, United Kingdom. Deidentified endo- and ectocervical tissue was collected from women undergoing therapeutic hysterectomy at St. Mary’s Hospital, London, United Kingdom. Penile and cervical tissue, including both epithelium and stroma, were cut into 3-mm^3^ explants, and four tissue pieces were added per well in a 24-well tissue culture plate. Multivirus competitions were set up such that each of the 11 Env_acute_ viruses was competed against the other and always against the three chronic viruses. Tissue blocks were then exposed to a mixture of four or five Env_acute_ viruses (B1, B2, B3, B4, B7, B8, B9, B14, B17, B19, and B20) and the same three Env_chronic_ viruses (I10, K44, and Q0) at 150 IU per virus for 3 h at 37°C. The tissue was then washed three times to remove free virus and cultured in complete RPMI 1640 medium (Sigma, United Kingdom) at 37°C.

Migratory cells (MC) migrating out of the tissue were harvested at 24 h and washed 3 times with phosphate-buffered saline (PBS) by centrifugation for 3 min at 350 × *g*. MC were then cocultured with 10^5^ PM1 CD4^+^ T cells in complete RPMI for 10 days at 37°C. The explant tissues were washed 3 times with PBS, resuspended in complete RPMI, and cultured for 10 days at 37°C. For MC–T cell cocultures and tissue cultures, half of the supernatant was harvested for analysis and replaced with fresh medium every 3 days. On day 10, the tissue was washed 3 times with PBS and lysed with 300 μl of 1% Triton X-100 supplemented with 1 mg/ml proteinase K overnight at 56°C. Cells from MC+PM1 T cell cocultures were washed 3 times with PBS and lysed with 300 μl of 1% Triton X-100. Each multivirus competition was performed in tissues from two donors in triplicate. DNA was extracted from lysates using the PureLink Pro 96 genomic DNA kit (Invitrogen, USA) according to the manufacturer’s instructions. The extraction procedure was performed using the epMotion 5075 automated liquid handling and pipetting system (Eppendorf, USA).

### HIV-1 transmission fitness assay in mannan-treated human explant tissue.

Transmission fitness assays were performed as described above with penile and cervical explant tissues incubated in medium alone or medium containing 1 mg/ml mannan (Sigma, Canada) for 12 h before infection with a mixture of one Env_acute_ (B4) and one Env_chronic_ (Q0) virus. Each condition was tested in tissues from three donors in triplicate. The total HIV-1 copy numbers in tissue and MC+PM1 cocultures were assessed using a Gag-specific qPCR.

### Flow cytometry.

For phenotypic analysis of DCs migrating from penile and cervical tissue, a multicolored flow cytometry antibody cocktail was used, consisting of CD14 Qdot605 (TüK4) (Invitrogen), CD11c AF700 (B-ly6), CD123 PECy5 (9F5), CD209 V450 (DCN46), Lin-1 fluorescein isothiocyanate (FITC), CD1c PECy7 (L161) (BioLegend, United Kingdom), HLA-DR allophycocyanin (APC) H7 (G46-6), CD207 APC (10E2) (BioLegend, United Kingdom), and CD83 phycoerythrin (PE) (HB15e). Unless otherwise specified, all antibodies were obtained from BD Biosciences, United Kingdom. Dead cells were excluded from analysis through staining with Aqua viability dye (Invitrogen, United Kingdom). Samples were acquired using an LSRIIFortessa fluorescence-activated cell sorter (FACS; BD Biosciences, United Kingdom) and analyzed using FlowJo (Tree Star, USA). Compensation matrices were created on FlowJo using single stained anti-mouse Ig(κ) negative-control compensation beads (BD Biosciences, United Kingdom).

### PCR and NGS analysis of multivirus infections.

HIV-1 DNA was PCR amplified from cellular DNA extracts. It is important to stress that the HIV-1 DNA and not the viral RNA was PCR amplified from cellular DNA extracts to ensure some level of virus infection rather than just residual inoculating virus after extensive washing. At a minimum, this viral DNA results from initial reverse transcription, but it likely represents a spreading HIV-1 infection due to the increasing viral DNA load over the 10-day incubation. PCR amplification of the C2-V3 envelope region and 454 sequencing were performed as previously described (6). The C2-V3 region of the envelope gene was amplified by an external-nested PCR amplification. Primers used for the external PCR were forward ENVB (5′-AGAAAGAGCAGAAGACAGTGGCAATGA-3′) (HXB2 positions 6202 to 6228) and reverse ED14 (5′-TCTTGCCTGGAGCTGCTTGATGCCCCAGAC-3′) (HXB2 positions 7932 to 7961), and those for nested PCR were forward E80 (5′-CCAATTCCCATACATTATTGTG-3′) (HXB2 positions 6858 to 6879) and reverse E125 (5′-CAATTTCTGGGTCCCCTCCTGAGG-3′) (HXB2 positions 7315 to 7338) (6). PCR mixtures contained a 0.2 μM concentration of each primer, 1.5 mM MgCl_2_, 1× Platinum *Taq* PCR buffer, 0.2 mM deoxynucleotide triphosphates (dNTPs), and 2 U Platinum *Taq* DNA polymerase. PCR cycle conditions were 95°C for 2 min, followed by 35 cycles of 95°C for 30 s, 55°C for 30 s, and 72°C for 2 min (external) or 45 s (nested), and a final extension of 72°C for 10 min.

To prepare the amplicon library for 454 sequencing, fusion primers including the Roche 454 Titanium key sequence, a multiplex identifier (MID) sequence for forward and reverse primers followed by the template-specific sequences forward E110 (5′-CTGTTAAATGGCAGTCTAGCAGAA-3′) (HXB2 positions 7002 to 7025) and reverse E125 (5′-CAATTTCTGGGTCCCCTCCTGAGG-3′) (HXB2 positions 7315 to 7338) were used. PCR cycle conditions were 95°C for 2 min, followed by 35 cycles of 95°C for 30s, 55°C for 30 s, and 72°C for 45 s, and a final extension of 72°C for 10 min. PCR products were run on a 1% agarose gel to verify the product size of 406 bp and purified using the Agencourt AMPure XP bead system with a bead-to-DNA ratio of 0.7:1 according to the Roche manual. Sample libraries were quantified using the Quant-iT PicoGreen double-stranded-DNA assay kit (Invitrogen, Canada), diluted, and pooled at 10^6^ molecules/μl for pyrosequencing. Emulsion PCR (emPCR) was performed with a ratio of 0.5 molecules of sample library per bead. Enriched beads (5 × 10^5^) were then loaded onto titanium picotiter plates according to the Roche 454 instructions. The sequencing run was performed on the Roche 454 GS Junior sequencer as previously described (6). Next-generation sequencing (NGS) analyses was employed as a sophisticated and sensitive counting tool, and the higher error rate seen with Roche 454 has no bearing on these results.

### Sequence data analysis.

A custom analysis pipeline (a revision of Segminator II [53] and SeekDeep [27]) was used to quantify each virus within a competition. First, raw sequence data were extracted by the MID tag. Next-generation sequences were analyzed for quality and length, and then the relative amount of each virus was determined via maximum-likelihood phylogenetic tree alignments, SeekDeep alignments, groups, and count sequences within a cluster aligning to each of the input viral sequences.

### Lectin binding assay.

The relative binding affinity of Env_acute_ and Env_chronic_ HIV-1 to C-type lectins was assessed using an Akubio RapID4 acoustic biosensor (TTP Labtech). DC-SIGN and langerin were prepared in sodium acetate buffer and covalently bound to a quartz crystal using an ethyl carbodiimide hydrochloride (EDC) and *N*-hydroxysulfosuccinimide (NHS) amine linkage. Free ester groups were neutralized by ethanolamine blocking. Viral stocks normalized for p24 concentration were allowed to flow over the immobilized lectins for 3 min at a flow rate of 25 μl min^−1^, followed by a 5-min dissociation step. The quartz crystal surface was regenerated using a 100 mM glycine buffer (pH 2.5) containing 0.05% Tween 20 for 3 min. PHA was used as a nonspecific lectin control.

### Immunofluorescence and imaging.

Frozen tissue sections (12 μm) were fixed in 3.7% formaldehyde in PIPES [piperazine-*N*,*N*′-bis(2-ethanesulfonic acid)] buffer for 5 to 10 min, followed by washing in cold PBS. Samples were blocked with normal donkey serum for 10 min and washed again. For mannan-binding lectin (MBL) identification, samples were stained (1:100; clone 3B6; Abcam) for 1 h at 37°C and then washed with cold PBS. Next, a Cy5-labeled donkey anti-mouse immunoglobulin secondary antibody (Jackson ImmunoResearch, USA) was added for ∼20 to 30 min at room temperature. Samples were washed, and Hoechst DAPI (4′,6-diamidino-2-phenylindole; Invitrogen, USA) was applied for 10 min to stain nuclei before a final wash with PBS. Mounting medium (DakoCytomation) and coverslips were applied to sections, sealed with clear nail polish, and stored at 4°C. Images were obtained by deconvolution microscopy on a DeltaVision RT system collected on a digital camera (CoolSNAP HQ; Photometrics) using a 40× oil objective. Panel images at a magnification of ×40 were acquired to include the epithelium and the underlying connective tissue in all *ex vivo* human samples. After image acquisition, SoftWoRx analysis software (Applied Precision) was used to stitch and quickly project up to 30 z-stacks per panel set.

### Quantitative PCR.

Samples were run using the QuantStudio5 real-time PCR system (Applied Biosystems, Canada) using *gag*-specific primers as previously described (54). First, an external HIV-1 *gag*-specific PCR was performed in a 50-μl reaction mixture containing 1× PCR buffer, 1 mM MgCl_2_, 0.2 μM dNTPs, 2 U of Platinum *Taq* DNA polymerase, and 0.2 μM concentrations of forward primer gag-cons1 (5′-GAGAGAGATGGGTGCGAGAGCG-3′) (HXB2 positions 783 to 804) and reverse primer UNIV-GA4 (5′-TTGCCAAAGAGTGACCTGAGGGAA-3′) (HXB2 positions 2250 to 2273). PCR was performed using the Simpliamp thermocycler (Applied Biosystems, Canada) under the following reaction conditions: 95°C for 2 min, followed by 35 cycles of 95°C for 30 s, 55°C for 30 s, and 72°C for 1 min 50 s, and a final extension of 72°C for 10 min.

Following PCR, 20 μl PCR product was added to a qPCR mixture which included 0.5 μM concentrations of forward primer gSCA F (5′-CATGTTTTCAGCATTATCAGAAGGA-3′) (HXB2 positions 1299 to 1323) and reverse primer gSCA R (5′-TGCTTGATGTCCCCCCACT-3′) (HXB2 positions 1359 to 1377), 0.2 μM gSCA probe (5′-FAM [6-carboxyfluorescein]-TACTGGGACAGCTACAACCATCCCTT-BHQ [black hole quencher]-3′) (HXB2 positions 968 to 993), and TaqMan Fast advanced master mix. Samples were run under the following reaction conditions: 60°C for 2 min, 95°C for 20 s, and 40 cycles of 95°C for 1 s and 60°C for 43 s. All samples were run in duplicate. Samples were considered negative when cycle threshold (*C_T_*) values were above 35.

### Statistical analysis.

Statistical analysis was performed on GraphPad Prism 6, using the Holm-Šidák test to compare the average replication of Env_chronic_ with Env_acute_ in each competition, a two-tailed Mann-Whitney test to compare the total average replication of Env_chronic_ and Env_acute_ in tissue (with and without mannan) with that in MC+PM1 cocultures, and a Spearman rank correlation analysis for comparison of lectin binding affinity and replication in tissue or MC+PM1 cocultures. *P* values of <0.05 were considered significant.
